# The short-term effect of a COVID-19 infection on employment probabilities of labour-market entrants in the Netherlands

**DOI:** 10.1007/s00181-025-02798-x

**Published:** 2025-08-11

**Authors:** Henri Bussink, Tobias Vervliet, Bas ter Weel

**Affiliations:** 1 SEO Amsterdam Economics, Amsterdam, The Netherlands; 2 Amsterdam School of Economics, University of Amsterdam, Amsterdam, The Netherlands

**Keywords:** COVID-19 infections, Employment, Young workers, J10, J23, I24

## Abstract

This research estimates the effect of a COVID-19 infection on the employment probabilities of two cohorts of labour-market entrants in the Netherlands. To identify the causal effect, we exploit variation in registered (positive) COVID-19 (PCR) test results among graduates over time and estimate a heterogeneity-robust difference-in-difference model. The empirical results suggest that a COVID-19 infection decreases the employment probabilities of positively tested labour-market entrants (ATT) by 0.5–1.1 percentage points over a three-month period within the first fifteen months after graduation. The effect size and duration are limited and predominantly driven by graduates from secondary vocational education and those who are just entering the labour market. The estimated coefficients for graduates from higher education and those who have already been employed for some months are economically small. Due to differences in group size and timing of the event, a direct comparison to the effect of lockdown measures is not possible. However, the effect size (ATT) seems to be at most ten percent of the average effect (ATE) of COVID-19 related lockdowns.

## Introduction

Graduating and entering the labour market during the COVID-19 pandemic has a negative effect on employment opportunities in the short run (Bussink et al. 2022).^1^ Limited employment opportunities and uncertainty during economic lockdowns have been the most likely causes disrupting labour supply and demand for graduates during the COVID-19 pandemic. Also infections with the COVID-19 virus (SARS-CoV-2) itself may have affected short-run employment probabilities.^2^ A COVID-19 infection can reduce employment opportunities of labour-market entrants if they become temporarily unavailable for work or are less productive while at work which could be damaging contract extension. Additionally, contracting COVID-19 and subsequently becoming ill or undergoing a period of quarantine can be particularly troublesome for those starting their job search. This is especially a burden when the nature of work is location-based and requires physical presence. As a result, it is possible that labour-market entrants who become infected may search less intensively, delay entering new job positions, and even have their job offers cancelled. Moreover, temporary contracts – which are the most common contracts labour-market entrants have been offered—may not be extended due to these circumstances. This leads to longer spells of job search and higher unemployment rates among graduates.

This research estimates the short-term effect of a COVID-19 infection on the employment probabilities of two cohorts of labour-market entrants in the Netherlands. We exploit the variation in (positive) COVID-19 test results registered at Dutch Municipal Health Services (GGD) between graduates over time to identify the causal effect of an infection on employment probabilities. We use the difference-in-difference (DiD) model developed by Callaway and Sant’Anna (2021). This model is robust to heterogenous and dynamic treatment effects in designs with multiple treatment periods, which contrasts the conventional approach based on the two-way fixed effects (TWFE) estimator (Roth et al. 2023; Arkhangelsky and Imbens 2023). Using this approach has value, because infections do not occur at the same time making the definition, composition and size of control and treatment groups less obvious than in a static treatment–control setting. Intuitively, we estimate weighted average treatment effects on the treated (ATT) exploiting all possible combinations of canonical two-period DiD designs that exists in the panel data between labour-market entrants who are first infected (which is proxied by a positive COVID-19 test result) in a particular month and those who are ‘never infected’, excluding those who are ‘not-yet infected’ as control units (according to the registration data at our disposal).

Additionally, we explore dynamic treatment effects over time by presenting event-study estimates of pre- and post-infection differences between infected and never-infected labour-market entrants. We also explore differences in dynamic treatment effects between secondary vocational (MBO) and higher education (HO) graduates by performing subsample analyses. The level of education is positively associated with remote-work opportunities during periods of quarantine (Barrero et al. 2023), which makes the split between education levels relevant. Finally, we assess the sensitivity of our estimated coefficients by using several alternative estimation procedures and by estimating alternative specifications of the control group and treatment variables.

The full sample consists of two cohorts of labour-market entrants between 16 and 30 years old. These people left education and entered the labour market in the Netherlands just before (2019 cohort) and during (2020 cohort) the COVID-19 pandemic. For each of the two cohorts, we analyse labour-market information for around 160,000 graduates from secondary vocational education (MBO) and higher education (HO). We only consider graduates who enter the labour market after completing their education by at latest the first of October after graduation, excluding continuing and returning students and those without a diploma or starting qualification (dropouts).^3^

To measure employment probabilities, we define employment as being employed as an employee for a minimum of three days a week (equivalent to a part-time work factor of 0.6). We combine total working hours from multiple concurrent jobs to determine if graduates work at least three days a week. Our analysis focuses solely on formally registered job positions, since data on informal employment or self-employment is unavailable or incomplete.

To measure COVID-19 infections, we define an infection as having obtained a positive COVID-19 test result, which is taken and/or registered at a public health service (GGD) test location. We have access to unique administrative data about test results for the period between June 2020 and December 2021. All graduates who have tested positively on a COVID-19 test at the GGD in the first months after entering the labour market are included in the treatment group. Once someone obtains a positive test result, he remains part of the treatment group in the following months. The control group consists of all graduates who never test (positively) at the GGD or whose test results are inconclusive or unknown in the first fifteen months after entering the labour market.^4^ During the COVID-19 pandemic there are many ways to take tests. We have only access to those people who have been registered at the public health service agency in the Netherlands. This is likely an underestimate of the real number of infected people. In terms of the empirical analysis, this leads to an underestimate of the effect of contracting COVID-19 on employment probabilities because it is likely that the control group also contains people who have contracted COVID-19, especially during periods with higher rates of COVID-19 cases when asymptomatic or untested infections were more common. Finally, we do believe that the data at our disposal provide the possibility of a scientifically sound analysis. Many (potential) workers who felt ill had to go to the public health service agency to take a test and were only able to work after having obtained a negative test result.

Our main estimates suggest that a COVID-19 infection decreases employment probabilities of labour-market entrants. Employment probabilities in the last month before a COVID-19 infection are on average 81.3–82.2 percent for the 2019 cohort and 78.3–79.8 percent for the 2020 cohort. For the 2019 cohort, a COVID-19 infection reduces the probability of employment by 0.5–0.7 percentage points on average. This effect is observed *after* the first year of graduation, specifically between 12 and 15 months after graduation, when both employment probabilities and COVID-19 test results are available in our data. For the 2020 cohort, a COVID-19 infection reduces the probability of employment by 1.0–1.1 percentage points on average. This effect is observed *within* the first year of graduation, specifically up to 11 months after graduation, when both employment probabilities and COVID-19 test results are available in our data. The difference in effect size between cohorts is likely due to differences in the period in which we are able to measure employment and infections, with the impact of COVID-19 infections likely being more pronounced on recent labour-market entrants compared to those who might already be employed.^5^

The findings further suggest that the effect of a COVID-19 infection on employment probabilities is relatively short-lived for the 2019 cohort, since only the estimates up to one month after having obtained a positive test result are statistically significant. For the 2020 cohort, the drop in employment probabilities persists for a longer period of time, with estimates being statistically significant up to three months after having obtained a positive test result. We do not observe any significant differences in employment probabilities during the pre-treatment period. This provides support for the parallel trends assumption (Roth et al. 2023). The findings also suggest that the drop in employment probabilities is larger for secondary vocational graduates than for higher education graduates. Moreover, for secondary vocational graduates, the drop persists for longer period of time, whereas for higher education graduates, it is relatively short-lived. A potential explanation is that the opportunity for remote work significantly increases with the level of education (Barrero et al. 2023). Sensitivity analyses demonstrate the robustness of our main findings to alternative estimation procedures and to alternative specifications of the control group and treatment variable.

Overall, the impact of contracting COVID-19 on employment probabilities of labour-market entrants in the Netherlands seems to be limited and temporary. Generally, it ranges from one to ten percent of the effect size of COVID-19 related lockdowns estimated by Bussink et al. (2022). Due to differences in estimation methods a precise comparison between these two studies is not possible, but the ballpark seems to be in this order of magnitude. As mentioned above, the effect of contracting COVID-19 on employment probabilities is likely to be underestimated in our approach because of the likely inclusion of infected individuals in the control group. This group is likely larger in periods with high infection rates, as more people may have been infected but remained untested and/or unregistered with the public health service agency. Finally, the larger estimated coefficients for graduates from secondary vocational suggest that a lower ability to work from home has negative employment effects when becoming infected with COVID-19.

This paper is organized as follows. Section 2 briefly discusses the related literature and the specific setting in which the analysis takes place. Section 3 describes the administrative data and methodology applied in this research. Section 4 presents the main empirical results and documents the estimated coefficients. Section 5 performs several sensitivity checks and shows that the results remain statistically significant across different model specifications. Section 6 provides a discussion of the main findings and concludes.

## Literature and setting

The COVID-19 pandemic has significantly disrupted the Dutch economy and its labour market. The first cases of COVID-19 (SARS-CoV-2) are diagnosed in February 2020. As infections surge across Europe, the Dutch government introduces a number of measures to curb the spread of the virus, including lockdowns, school closures, travel restrictions, distancing measures and stay-at-home orders. The spread of COVID-19 progresses in multiple waves, while the government’s response evolves through distinct policy phases reflecting changing mitigation strategies (e.g. Van Amerongen et al. 2024).

The first phase (February 2020-January 2021) relies on non-pharmaceutical interventions (NPIs), with the first wave (March-June 2020) triggering the first almost complete economic lockdown. Following a temporary reduction in cases, the second wave emerges (from late 2020 to early 2021), leading to renewed restrictions, including the second full lockdown. During this phase, containment measures focus on social distancing, remote-work recommendations, and gradual expansions in healthcare capacity to manage the surge in hospitalizations.

During the second phase (January 2021—January 2022) vaccines become available, while maintaining stringent NPIs. Although vaccination efforts start in early 2021, initial hesitancy and logistical challenges delay widespread immunization. The third wave (late 2021) coincides with increasing vaccination rates but also requires further restrictions, including another partial lockdown, due to the emergence of new variants of the virus, such as Delta and Omicron. The government adapts containment measures in response to fluctuating infection rates and hospitalizations, gradually adjusting restrictions as vaccine coverage expand.

The third phase (January 2022—February 2023) marks the post-vaccine and booster period with minimal or absent NPIs as immunity increases. With a significant portion of the population vaccinated and booster campaigns underway, the healthcare system stabilizes, allowing most COVID-19-specific restrictions to be lifted by mid-2022. Although new infections still occur, hospitalizations and mortality rates remain relatively low, leading to a transition towards an endemic approach to managing COVID-19. Economic indicators reflect these three phases, with GDP contracting significantly in 2020, followed by gradual recovery in 2021–2022 and labour-market tightness due to high levels of labour demand.

To mitigate economic consequences and damage, the government launches several financial support programmes, such as wage subsidies, compensation and tax deferrals for affected businesses. Despite these policies, the COVID-19 pandemic has led to a fall in employment and hours worked in 2020 in the Netherlands. Although the financial support has reduced the impact of economic lockdowns to a large extent, some labour-market groups have been adversely affected. In particular, young people and workers on temporary contracts are most severely affected by economic lockdowns. The number of registered job seekers (aged 15–27) increases from 111.000 to 134.000 persons in 2020. The number of unemployed persons aged 15–25 increases from 143.000 to 175.000 persons in 2020, whereas total unemployment rises from 423.000 to 465.000 persons in the same period. Most of the burden seems to be on young workers, who face short periods (up to six months) of unemployment during economic lockdowns.

Despite this relatively large impact of the COVID-19 pandemic on youth employment, evidence so far does not point towards severe adverse effects on labour-market entrants with a diploma (Bussink et al. 2022). For those leaving school with a diploma by October 2019 and 2020, employment rates a year after graduating are more or less similar to those who finished education before the pandemic. Of course, these labour-market entrants have had more trouble finding jobs and some seem to have postponed labour-market entrance (both forced because it was impossible to fulfil formal requirements to graduate and voluntarily because of economic uncertainty), which has led to short-term adverse effects on employment (e.g. Bussink et al. 2022 for the Netherlands, Casarico & Lattanzio 2022 for Italy, Churchill 2021 for Australia, and Joyce & Xu 2020 for the United Kingdom). In addition, it remains unclear whether the quality of employment is the same compared to earlier cohorts and to what extent graduates have been adversely affected by school closures in terms of benefitting from human capital investments (Jakubowski et al 2024).^6^

This research contributes to the emerging literature on the impact of the COVID-19 pandemic on socio-economic and labour-market outcomes by focussing on the labour-market effects of contracting COVID-19. International research on the macroeconomic effects of the COVID-19 pandemic shows that employment losses have been significant due to the pandemic and its measures, and that the magnitude and duration vary greatly between countries and different groups of workers (Adams-Prassl et al. 2020; Chetty et al. 2020). In the Netherlands, the overall effect of the COVID-19 crisis on the labour market was shorter-lived and less severe than during an economic recession because of generous financial support packages for businesses (Bussink et al. 2022). This seems to be the case in many countries, e.g. United Kingdom and Australia for similar work (Joyce & Xu 2020; Churchill 2021).

What remains unclear is the labour-market effect of contracting COVID-19. We contribute to the up to now scarce literature that directly relates COVID-19 infections to socio-economic and labour-market outcomes at the individual level, with a particular focus on labour-market entrants. International research based on survey data indicates a relation between COVID-19 infections and (prolonged) sick leave, leading to a substantial reduction in the number of hours worked. This literature mainly focusses on severe cases of infections and infections that cause long periods of leave. Ziauddeen et al. (2022) highlight the heterogeneity of persistent symptoms, and the significant functional impact of prolonged illness following confirmed or suspected COVID-19 infections. Davis et al. (2021), Cutler (2022) and Evans et al. (2022) show that even after several months patients with long COVID or hospital admissions have not-yet recovered, have not returned to previous levels of work, and continue to experience a significant symptom burden and a reduced health-related quality of life. Ham (2022) specifically investigates the relationship between long-haul COVID and labour-market outcomes and finds that long-haul COVID patients are more negatively impacted in terms of employment status and number of hours worked than similar individuals who have only mild symptoms. Finally, an U.S. event study suggests that COVID-19-related absence (of at least one week) is likely to reduce total labour supply by approximately 0.2 percentage points (equivalent to 500,000 workers at the macroeconomic level) (Goda and Soltas 2023).

Evidence on labour-market effects for young workers and labour-market evidence seems absent from this research. Snape and Viner (2020) present an overview of the medical literature which suggests that children and adolescents on average face relatively mild symptoms when contracting COVID-19. One of the reasons could be that young workers are more likely to be normal-weight persons than older persons. People with obesity are more likely than normal-weight people to have other diseases that are independent risk factors for severe COVID-19, including heart disease, lung disease, and diabetes. These other diseases have shown to be correlated with severe COVID-19 symptoms (Popkin et al. 2020). In general, it seems to be the case that a better health status is on average negatively correlated with severe COVID-19; and young people seem to be in better health on average compared to older workers.

## Data and methodology

This section presents the most salient details of the data at our disposal. It also documents a number of trends to show that the models we are applying are valid. We end by presenting the empirical strategy to show the effect of COVID-19 infections on employment probabilities.

### Data and treatment assignment

All administrative data sources have been accessed by making use of remote-access facilities at Statistics Netherlands. To conduct the research, we use several data sources about which we report in this section and of which the data on infections have been made available for this research.

We use administrative data from Statistics Netherlands on educational enrolment and degrees to identify graduates between 16 and 30 years old who left education and entered the labour market in the Netherlands just before (2019) and during (2020) the COVID-19 pandemic. The data are registered at the Dutch Education Executive Agency (DUO) and made available by Statistics Netherlands. For each of the two cohorts, we have obtained labour-market information for around 160,000 graduates at different levels of education. We restrict our sample to individuals entering the labour market after completing their education and obtaining a diploma by at latest the first of October after their year of graduation, and who are considered to be available for work. We distinguish between graduates from secondary vocational education (MBO) and higher education (HO). We exclude individuals who continue studying after completing a certain level of education, those who return to education within a year, and those who enter the labour market without a diploma or starting qualification (dropouts)—see Feron et al. (2016) and Bisschop et al. (2020) for a more detailed description of the Dutch education system, in particular about vocational and higher education tracks.

To measure employment probabilities, we use administrative data on employer-employee relations (contracts, wages and allowances) from the Dutch Employee Insurance Agency (UWV) and the Dutch Tax Administration, which is made available by Statistics Netherlands. We define employment as having a job as an employee for a minimum of three days per week (equivalent to a part-time work factor of 0.6). If someone has multiple jobs concurrently, the total working hours are combined to determine whether they are working at least three days a week. Our analysis only considers formally registered job positions as an employee, since information regarding informal employment or self-employment is not available or incomplete in the data at our disposal.

To measure COVID-19 infections, we use administrative data on COVID-19 test results from the national organization of Municipal Health Services and Regional Medical Assistance Organizations (GGD GHOR), which is made available by Statistics Netherlands specifically for conducting this research. These data come from two different data sources: CoronIT and HPZone Lite. The first data source (CoronIT) includes the results of all PCR tests^7^ that have been taken and have been registered at a Municipal Health Service (GGD) test location. The results of the PCR tests can be (presumably) positive, negative, inconclusive or unknown. The second data source (HPZone Lite) includes only positive test results of all PCR tests that have been taken and have been registered at a GGD test location, and also positive test results that have been reported to the GGD by external doctors and medical microbiology laboratories, but that are not registered at CoronIT (which are for 99.8 percent also PCR tests).^8^ The second data source has a slightly larger representation of COVID-19 tests than the first data source, but misses data on negative test results instead.

A labour-market entrant is allocated to the treatment group if at least one COVID-19 test is positive within the first fifteen months after graduation (see Table 1), while only those who have never tested (positive) are allocated to the control group, excluding those who have not-yet tested (positive) as control units. In our main analysis, an individual who has tested positively remains part of the treatment group for the entire observation period. We utilize all test results registered between June 2020 and December 2021 at the GGD. It is important to note that the availability and use of COVID-19 tests evolves over time, which may influence the definition of treated and control units across cohorts. For the 2019 cohort, test availability is initially limited, with free testing becoming gradually accessible. For the 2020 cohort, testing is widely available from the start of the observation period. This difference may affect the likelihood of being classified as infected, potentially leading to differences in the composition of treatment and control groups between the two cohorts.

**Table 1:** Sample of COVID-19 (PCR) test results. *Source:* Statistics Netherlands, GGD CoronIT and HPZone Lite

	Cohort 2019		Cohort 2020	
Data source	CoronIT	HPZone Lite	CoronIT	HPZone Lite
Observation period	9–15 months	9–15 months	1–11 months	1–15 months
Treatment group				
Tested and positive	9,427	10,474	23,293	37,756
Control group				
Tested and negative	57,836	0	71,859	0
Tested and inconclusive or unknown	245	97	1,058	263
No test reported	92,878	149,815	57,504	115,695
No. of graduates	160,386	160,386	153,714	153,714

The table reports the number of graduates from the 2019 and 2020 cohort that are allocated to the treatment or control group according to COVID-19 (PCR) test results based on the CoronIT and HPZone Lite data. The treatment group includes all graduates with at least one observed positive COVID-19 (PCR) test result within the first fifteen months after graduation. The control group includes all graduates for whom we never observe a (positive) COVID-19 (PCR) test result or whose test results are inconclusive or unknown in the first fifteen months after graduation

Limited data availability means that we only observe COVID-19 test results between the ninth and fifteenth month after graduation of the 2019 cohort who enter the labour market in October 2019. The ninth month after graduation corresponds to June 2020, the first month that free COVID-19 tests becomes available at the GGD for healthcare workers and educational staff. The fifteenth month after graduation corresponds to December 2020, when free COVID-19 tests are available at the GGD for all people with symptoms of contracting COVID-19. For the 2020 cohort, we observe COVID-19 test results from the first month after graduation onwards, which corresponds to October 2020. At that time COVID-19 tests are already widely available for five months for healthcare workers and educational staff and for three months for all people with COVID-19 symptoms.

**Fig. 1:**
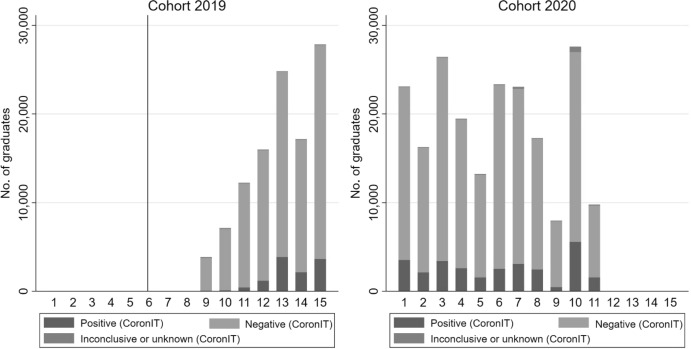
All COVID-19 (PCR) test results by cohort and month after graduation (based on CoronIT). *Note*: The figure displays the development of all COVID-19 (PCR) test results (positive, negative, inconclusive or unknown) for the 2019 and 2020 cohort by month after graduation (1–15 months) based on CoronIT data. The reference line (sixth month after graduation for the 2019 cohort) corresponds to the start of the COVID-19 pandemic in the Netherlands (March 2020). For months with missing bars, there is no data on COVID-19 (PCR) test results available in the data at our disposal. *Source*: Statistics Netherlands, based on GGD CoronIT

Figure 1 shows the development of COVID-19 test results for the 2019 and 2020 cohort by month after graduation. For the 2019 cohort, the number of COVID-19 tests increases from less than 5,000 in June 2020 (nine months after graduation), when testing at the GGD is at its infancy, to almost 25,000 in December 2020 (fifteen months after graduation), when testing becomes more common. The proportion of positive tests varies between 1 and 16 percent. During this period, the average employment probabilities of this cohort of graduates starts recovering after the first lockdown (March-June 2020) to pre-COVID-19 levels, following a decline of up to 4 percentage points from an initial level of 77 percent (Bussink et al. 2022). For the 2020 cohort, the number of COVID-19 tests varies in the first eleven months after graduation (from October 2020 to August 2021) between 7000 and 23,000, with 6 to 20 percent resulting in a positive result. During this period, the average employment probabilities of this cohort of graduates experiences only a minor decline and quickly recovers to pre-COVID levels of approximately 77 percent (Bussink et al. 2022).

**Fig. 2:**
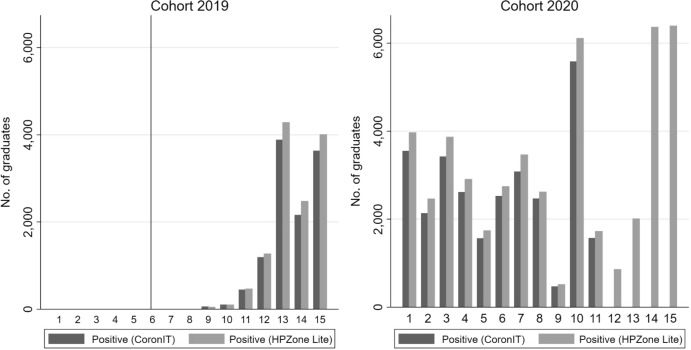
Only positive COVID-19 (PCR) test results by cohort and month after graduation (CoronIT versus HPZone Lite). *Note*: The figure displays the development of only positive COVID-19 (PCR) test results for the 2019 and 2020 cohort by month after graduation (1–15 months) based on HPZone Lite data. The reference line (sixth month after graduation for the 2019 cohort) corresponds to the start of the COVID-19 pandemic in the Netherlands (March 2020). For months with missing bars, there is no data on COVID-19 (PCR) test results available in the data at our disposal. *Source*: Statistics Netherlands, based on GGD CoronIT and HPZone Lite

Figure 2 shows the test results from the HPZone Lite data. This database contains positive test results only. The pattern is similar to the pattern in the CoronIT database. The advantage of the HPZone Lite database is that it also allows us to measure COVID-19 infections up to fifteen months after graduation for the 2020 cohort (up to December 2021).

### Summary statistics and parallel trends

The empirical analysis requires that control and treatment groups are similar. To show the likelihood of this similarity, Tables 5 and 6 in the Appendix display the differences in a number of observable characteristics of infected and never-infected individuals in both cohorts based on our two data sources of COVID-19 test results. In general, the statistics suggest that a COVID -19 infection (i.e. a positive COVID-19 test result) seems to be more likely among women (0.7 to 4.7 percentage points) and graduates with a migrant background (2.4 to 7.3 percentage points). Graduates who are higher educated (-3.0 to 0.3 percentage points) and have higher educated parents (-1.8 to -2.5 percentage points) seem to be less likely to have contracted COVID-19. Finally, living with housemates or family members (-1.5 to 1.8 percentage points), and having fewer health issues (-0.8 to 0.8 percentage points) are either positively or negatively correlated with contracting COVID-19, depending on the cohort and data source applied. These differences between the control and treatment group are important to take into account in the empirical strategy, as they may influence both the likelihood of infection and employment outcomes. Failing to account for these factors could lead to biased estimates of the effect of COVID-19 on employment probabilities. Since testing positive is positively related to having a job (2.0 to 3.7 percentage point, see Tables 5 and 6), for example, because employers require employees to be tested before showing up at work, the effect of contracting COVID-19 on the probability of employment is likely to be biased in such a way that it reverses causality. In addition, individuals who are living with housemates or family members and who do not shy away from physical social activities, including going to work, might be more likely to contract COVID-19. If individuals sort according to risk preferences, it could be the case that those living with housemates or family members and regularly attending physical social activities are systematically different from individuals living on their own and who do not regularly take part in such activities. Finally, exposure to the COVID-19 virus is likely to also be associated with a range of other socio-economic factors. While systematic differences in levels between infected and never-infected graduates pose no immediate threat to the internal validity of our empirical strategy, we may still be concerned that differences could be associated with diverging trends in employment probabilities between the control and treatment groups.

To mitigate this concern, we apply a generalized difference-in-difference approach with individual fixed effects, which accounts for any individual differences that are constant over time. Rather than including covariates directly in the model, we use the base-period levels of the background characteristics of Tables 5 and 6 to balance the distribution of covariates between treated and control individuals based on inverse probability tilting. The identifying assumption is the assumption of conditional parallel trends, which implies that the infected and never-infected individuals would follow common trends in employment probabilities in the absence of a positive COVID-19 test result and conditional on background characteristics. This assumption generalizes the standard parallel trends assumption, which requires that treatment and control groups would have experienced the same trends irrespective of individual characteristics. The conditional variant relaxes this by allowing differences in levels and trends across individuals, as long as these are fully captured by the observed covariates. This makes the assumption more plausible in settings where treatment may be related to background characteristics. To assess the credibility of this assumption, we exploit the presence of multiple treatment periods and plot pre-infection trends for each treatment group, comparing them to the trend in employment probabilities in the control group during the same period of time.

**Fig. 3:**
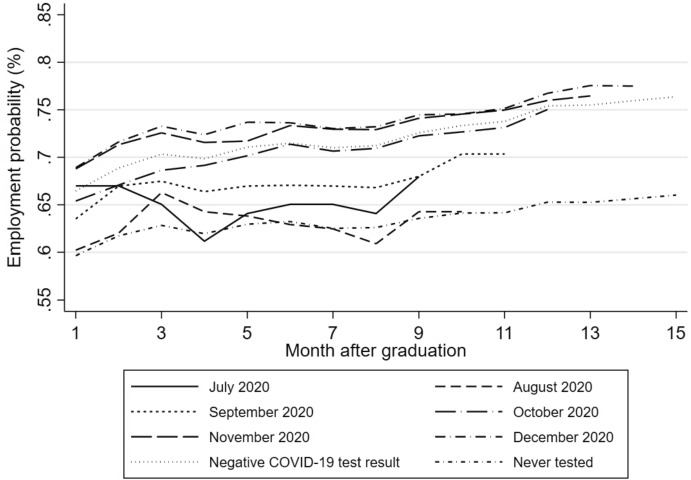
Pre-infection development of employment probabilities of the 2019 cohort by month after graduation and month of positive COVID-19 test result (based on CoronIT). *Note*: The figure displays the pre-infection development of employment probabilities of the 2019 cohort by month after graduation (1–15 months) and month of the first positive COVID-19 (PCR) test result (July 2020 till December 2020) based on CoronIT data. The pre-trend for the control group is reflected by the group with a negative, inconclusive or unknown COVID-19 (PCR) test result and those who never tested. *Source*: Statistics Netherlands, based on GGD CoronIT

Figures 3 and 8 (2019 cohort) and 4 and 9 (2020 cohort) show that in both cohorts almost all treatment groups follow a similar trend in employment probabilities in the period before treatment, albeit at different levels of employment, compared to the same period for the control group, even without conditioning on background characteristics. The control groups contain individuals with a negative COVID-19 test result and those who never tested, which are separately displayed in the figures. Only those from the 2019 cohort who test positively in July and August 2020 show a somewhat different employment trend. Since these months are the very first few months of testing and these groups only reflect a small number of individuals (between 46 and 355), we exclude them from the statistical analyses. In Sect. 5, we present the results of several sensitivity tests exploring the similarity of trends prior to a COVID-19 infection between infected and never-infected individuals.

### Empirical strategy

To analyse the short-term effect of a COVID-19 infection on the employment probabilities of labour-market entrants, we estimate a difference-in-difference (DiD) model. With multiple treatment periods, the conventional DiD approach is to estimate a static or dynamic two-way fixed effects (TWFE) model. The static TWFE specification would regress the employment probability on individual and time fixed effects and an indicator for whether or not graduate $i$ is infected in month $t$:

1 $$ Y_{i,t} = \alpha_{i} + \phi_{t} + D_{i,t} \beta_{post} + \varepsilon_{i,t} $$

The dynamic TWFE specification replaces the single infection indicator by dummies for months relative to the first positive COVID-19 (PCR) test result:

2 $$ Y_{i,t} = \alpha_{i} + \phi_{t} + \mathop \sum \limits_{r \ne 0} 1\left[ {R_{i,t} = r} \right]\beta_{r} + \varepsilon_{i,t} $$

However, recent literature shows the potential bias arising in generalized DiD settings with multiple treatment periods and heterogenous treatment effects (Goodman-Bacon 2021; Baker et al. 2022; de Chaisemartin and D’Haultfoeuille 2020, 2022, 2023; Arkhangelsky and Imbens 2023). In the case of variation in treatment timing and treatment effects, the conventional DiD approach based on the TWFE estimator can potentially result in biased and sometimes even misleading estimates. This might be due to the problem of ‘forbidden comparisons’ and the corresponding risk of ‘negative weights’. The problem arises in our research if earlier infected individuals are used as controls for later infected individuals, who cannot be explicitly ruled out using conventional TWFE estimation (see e.g. Roth et al. 2023 for a discussion).

To avoid such problems, we use the DiD approach developed by Callaway and Sant’Anna (2021), which is robust to heterogenous treatment effects in designs with multiple treatment periods. Moreover, this approach is preferred relative to alternative estimators that aggregate heterogeneous treatment effects in settings with staggered treatment timing because it accounts for serial correlation in the outcome and addresses concerns about the validity of parallel trends over longer time horizons (Roth et al. 2023).^9^ The approach first aggregates individuals into groups that are infected for the first time in the same month. It then estimates all group-time average treatment effects ($ATT\left(g,t\right)$), which are the average treatment effects on the treated (ATT) for each specific group $g$ measured at time $t$. Intuitively, the approach estimates all possible combinations of canonical two-period DiD designs that exist in the panel data. Since we rely on an imbalanced panel of individuals (because some individuals leave the Netherlands or die over time), only observations that are balanced within a given two-period DiD design are used for estimation.

The model of Callaway and Sant’Anna (2021) looks as follows:

3 $$ ATT\left( {g,t} \right) = E\left( {Y_{i,t} - Y_{i,g - 1} |G_{i} = g} \right) - E\left( {Y_{i,t} - Y_{i,g - 1} |G_{i} = G_{control} } \right) $$

where $ATT\left(g,t\right)$ represents the group-time average treatment effects,$E\left({Y}_{i,t}-{Y}_{i,g-1}|{G}_{i}=g\right)$ refers to the expected difference in outcome for group $g$ between time $t$ and the month before the infection occurs ($g-1$) and $E\left({Y}_{i,t}-{Y}_{i,g-1}|{G}_{i}={G}_{control}\right)$ is the expected difference in outcome for individuals who are ‘never infected’. By only using ‘never infected’ individuals and not ‘not-yet infected’ individuals as the control group, we avoid the forbidden comparisons problem, and hence the risk of negative weights (Roth et al. 2023). However, the diagnostic approach proposed by de Chaisemartin and D’Haultfoeuille (2020) shows that the number/fraction of group-time ATT’s that receive negative weights with static TWFE estimation is zero in our case. Additionally, $ATT(g,t)$ cannot be estimated for ‘always infected’ graduates, excluding graduates who were treated in the first month of the period in which we observe COVID-19 test results. To provide a meaningful interpretation of these estimates, we aggregate the individual group-time average treatment effects to provide weighted average treatment effects:

4 $$ ATT = \frac{{\sum w_{g,t} ATT\left( {g,t} \right)}}{{\sum w_{g,t} }} $$

where ${w}_{(g,t)}$ are weights based on the number of treated observations in a particular $ATT(g,t)$ (Roth et al. 2023). We use the improved doubly robust estimator based on inverse probability of tilting, which balances the distribution of the base-period levels of covariates between treatment and control group (see Tables 5 and 6 in the Appendix), and weighted least squares, which gives more weight to larger and more precise estimators than those derived from fewer observations (Sant’Anna and Zhao, 2020). Robust and asymptotic standard errors are computed and clustered at the individual level.

Following Eq. (4), we provide three aggregations of the group-time average treatment effects. First, we estimate a weighted average of all group-time average treatment effects with weights proportional to group size, to provide an overall ATT. Second, we estimate group-specific average treatment effects, which generates an ATT for all treatment groups. Finally, we estimate time-specific average treatment effects, which produces an ATT for all calendar months. We use six observations before ($t-1,\dots ,t-6$) and after ($t=0,\dots ,t+3$) the first month of a positive COVID-19 test result in the following analyses.^10^

## Results

This section documents and interprets the estimated coefficients. It starts with the average treatment effects on the treated (Sect. 4.1) and continues with dynamic treatment effects over time (Sect. 4.2). Finally, a subsample analysis based on different levels of education is presented in Sect. 4.3.

### Average treatment effects on the treated

We present average treatment effects on the treated (ATT) obtained from the aggregation of group-time average treatment effects (see Eq. 4) based on our definition of a COVID-19 infection and our measurement of employment probabilities. The average is calculated over a three-month period after graduation. A COVID-19 infection is defined as having obtained a positive COVID-19 test result taken and/or registered at a public health service (GGD) test location. Employment probabilities are defined as being employed as an employee for at least three days a week (equivalent to a part-time work factor of at least 0.6). The estimated coefficients are displayed in Panel A of Table 2. The average employment probability in the last month before a COVID-19 infection ($g-1$) is 81.3 to 82.2 percent for the 2019 cohort and 78.3 to 79.8 percent for the 2020 cohort.

**Table 2:** Main results. *Source:* Statistics Netherlands, based on GGD CoronIT and HPZone Lite

	Cohort 2019		Cohort 2020	
	(1) CoronIT	(2) HPZone Lite	(3) CoronIT	(4) HPZone Lite
Employment probability ($g-1$)	0.813	0.822	0.783	0.798
A. ATT	-0.007*** (0.003)	-0.005* (0.003)	-0.011*** (0.002)	-0.010*** (0.001)
B. ATT by group	-0.005** (0.002)	-0.004 (0.002)	-0.011*** (0.002)	-0.010*** (0.001)
C. ATT by time	-0.012*** (0.004)	-0.008** (0.003)	-0.013*** (0.002)	-0.011*** (0.002)
Pre-trend (p-value)	0.337	0.308	0.777	0.899
No. of graduates	159,900	159,883	150,689	150,295
No. of observations (graduate-months)	1,119,300	1,119,181	1,593,275	2,022,197

The table reports different aggregations of the group-time average treatment effects (ATT) of a COVID-19 infection on the employment probabilities of graduates from the 2019 (columns 1–2) and 2020 (columns 3–4) cohort. The coefficients are estimated using the heterogeneity-robust DID estimator by Callaway and Sant’Anna (2021) and are based on data on COVID-19 (PCR) test results from the CoronIT (columns 1 and 3) and HPZone Lite (columns 2 and 4) database. Panel A reports the overall ATT. Panel B reports the group-specific ATT, where the groups are defined by the month of the first positive COVID-19 (PCR) test result. Panel C reports the time-specific ATT, where time is defined by the calendar-month since graduation. Standard errors clustered at the individual level in parentheses; *p < 0.1, **p < 0.05, ***p < 0.01

The estimated coefficients suggest that a COVID-19 infection decreases the employment probabilities of labour-market entrants. For the 2019 cohort, a COVID-19 infection reduces the probability of employment by 0.5–0.7 percentage points (columns (1) and (2)) on average. This effect is observed *after* the first year of graduation, specifically between 12 and 15 months after graduation, when both employment probabilities and COVID-19 test results are available. The estimated coefficient based on CoronIT data is statistically significant at a 1 percent significance level. For the 2020 cohort, a COVID-19 infection reduces the probability of employment by 1.0–1.1 percentage points (columns (3) and (4)) on average. This effect is observed *within* the first year of graduation, specifically up to 11 months after graduation, when both employment probabilities and COVID-19 test results are available. These estimated coefficients are statistically significant at a 1 percent significance level.

The difference in effect size between cohorts is likely due to differences in the observation period. For the 2019 cohort, we relate a positive COVID-19 test result to employment probabilities starting from the twelfth month after graduation, whereas for the 2020 cohort, the observation window only extends until the eleventh month after graduation. Since employment probabilities for labour-market entrants generally increase over time, it seems likely that a COVID-19 infection has a larger impact on employment probabilities for those who are still in the early stages of job searching compared to those who may have already found a job and contract COVID-19 while working.

However, other factors beyond differences in time since graduation may have contributed to variations in effect size between cohorts. First, labour-market conditions evolve over time following the outbreak of the pandemic. The COVID-19 crisis may have influenced employment probabilities through multiple channels, including reduced employment opportunities (Bussink et al. 2022), shifts in hiring practices, and lower job search efforts (Balgová et al. 2022), particularly among discouraged job seekers. The pandemic also altered sectoral demand, with some industries experiencing more severe disruptions than others, which may have affected employment prospects differently for the two cohorts. Although the 2019 cohort is observed later after graduation—when employment probabilities are generally higher—they are exposed to a more volatile and disruptive phase of the pandemic. In contrast, the 2020 cohort is observed earlier after graduation, but under a more stabilized and better-adjusted phase of the pandemic with gradually improving conditions. These differences between the observation period and the phase of the pandemic may have offsetting effects on the estimated impact of infection.

Second, differences in the spread and severity of COVID-19 across time may have influenced the estimates. The incidence of infections varies between pandemic waves, affecting factors such as illness duration, hospitalization rates, and mortality (Van Amerongen et al. 2024). While our analysis does not directly capture these variations and young workers seems to be less affected, they could impact the labour market in different ways, for instance, through increased work absences, greater economic uncertainty, or changing employer behaviour. These factors may have influenced the extent to which a COVID-19 infection disrupted job search or employment, potentially leading to larger estimated effects for individuals infected during more severe waves. As such, variation in the epidemiological context may partly account for differences in effect sizes across cohorts.

Third, COVID-19 testing availability and practices have changed significantly between the two cohorts (Van Amerongen et al. 2024). For the 2019 cohort, test availability is initially limited, with free testing becoming gradually accessible. By contrast, for the 2020 cohort, testing is widely available from the start of the observation period onwards. This difference may influence the classification of treatment and control groups, as individuals with mild or asymptomatic infections are more likely to go undetected in the earlier period. Consequently, the control group for the 2019 cohort may contain a larger share of undiagnosed cases, leading to a larger underestimation of the true effect of infection on employment. This misclassification issue is particularly relevant during periods of high infection rates when untested infections are more common.

Finally, additional labour-market dynamics may have played a role. The ability to work remotely during a period of quarantine, which varies by sector and job type, may have mitigated employment disruptions for some individuals but not for those still in the job search phase (e.g. Flisi and Santangelo 2022). Similarly, the feasibility of conducting job interviews remotely may have affected job search outcomes. Although our data do not allow to measure these factors directly, they should be considered when interpreting our estimated coefficients.

**Fig. 4:**
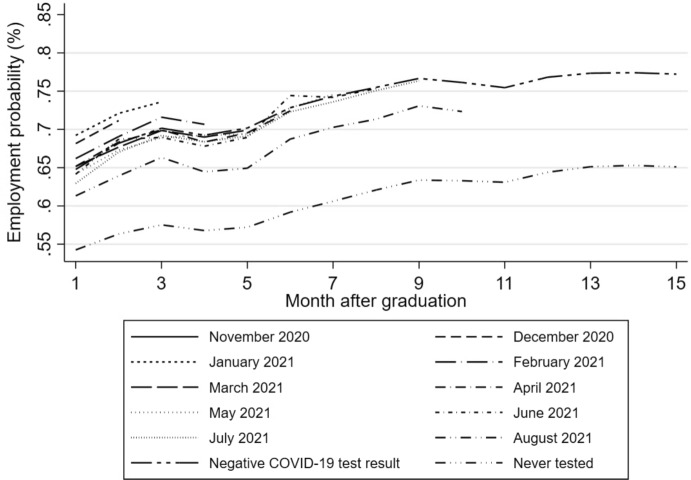
Pre-infection development of employment probabilities of the 2020 cohort by month after graduation and month of positive COVID-19 test result (based on CoronIT). *Note*: The figure displays the pre-infection development of employment probabilities of the 2020 cohort by month after graduation (1–15 months) and month of the first positive COVID-19 (PCR) test result (November 2020 till August 2021) based on CoronIT data. The pre-trend for the control group is reflected by the group with a negative, inconclusive or unknown COVID-19 (PCR) test result and those who never tested. *Source*: Statistics Netherlands, based on GGD CoronIT

The decomposition of the aggregate ATT into an ATT by group (row B) and by time (row C) shows no significant differences between the models. However, the above-mentioned factors highlight that differences in effect size between cohorts may not only reflect differences in time since graduation but also broader economic, epidemiological, and institutional changes that shaped employment outcomes during this pandemic.

### Dynamic treatment effects

**Fig. 5:**
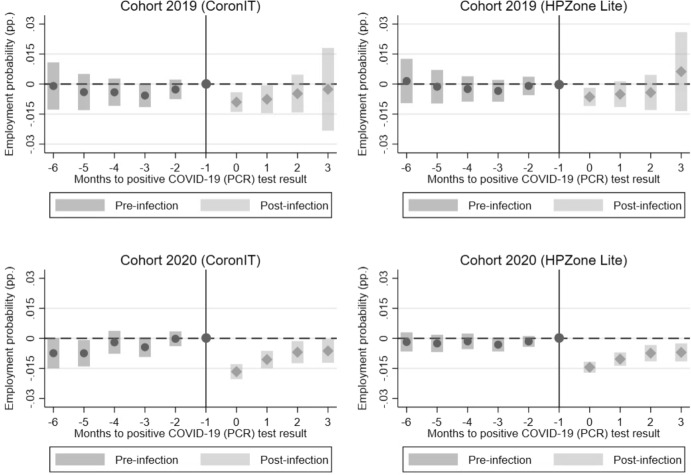
Event-study estimates of ATT. *Note*: The figure displays the event-study estimates (dots) and 95% confidence intervals (bars) of the ATT relative to the month of the first positive COVID-19 (PCR) test result for the 2019 and 2020 cohort based on CoronIT and HPZone Lite data. Period g-1 is used as the base-period and t as the post-period for ATT’s before (pre-infection) and after (post-infection) the infection takes place. *Source*: Statistics Netherlands, based on GGD CoronIT and HPZone Lite

We next investigate dynamic effects of a COVID-19 infection on employment probabilities of labour-market entrants over time, across the months after a positive COVID-19 test result. Specifically, we estimate pre-infection differences up to six months before a positive COVID-19 test result ($t-1,\dots ,t-6$), the instantaneous effect ($t=0$) and post-infection effects up to three months after a positive COVID-19 test result ($t+1,\dots ,t+3$). The last month before the infections takes place ($g-1$) is used as the base-period. These event-study estimates are displayed in Fig. 5.

The estimated coefficients suggest that the effect of a COVID-19 infection on employment probabilities is relatively short-lived for the 2019 cohort, since only the estimates up to one month after a positive test result are statistically significant. For the 2020 cohort, the drop in employment probabilities persists for a longer period of time, with estimates being statistically significant up to three months after a positive test result. We do not observe any significant differences in employment probabilities during the pre-treatment period between control and treatment groups. This supports the plausibility of the identifying assumption. As we use a doubly robust estimator based on inverse probability tilting to balance baseline covariates, the relevant assumption is that of conditional parallel trends. The absence of pre-treatment differences strengthens our confidence that, conditional on observed characteristics, treated and control groups would have followed similar trends in the absence of infection. Overall, the results are consistent with the DiD-estimates that contracting COVID-19 seems to temporarily disrupt employment probabilities of recent labour-market entrants.

### Differences by level of education

**Fig. 6:**
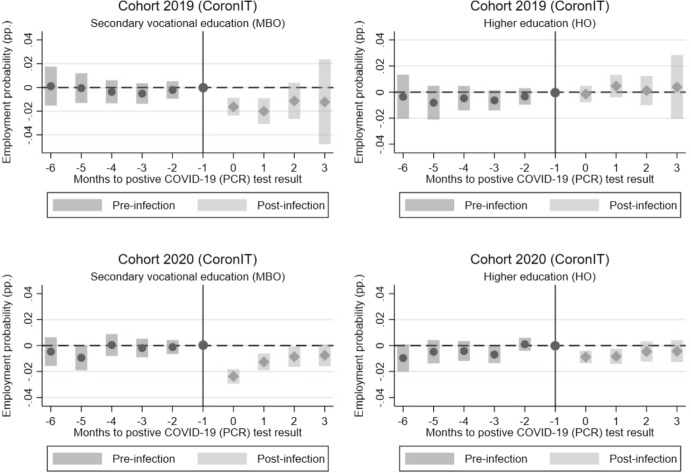
Event-study estimates of ATT by level of education (based on CoronIT). *Note*: The figure displays the event-study estimates (dots) and 95% confidence intervals (bars) of the ATT relative to the month of the first positive COVID-19 (PCR) test result for the 2019 and 2020 cohort based on CoronIT data. Period g-1 is used as the base-period and t as the post-period for ATT’s before (pre-infection) and after (post-infection) the infection takes place. *Source*: Statistics Netherlands, based on GGD CoronIT

**Fig. 7:**
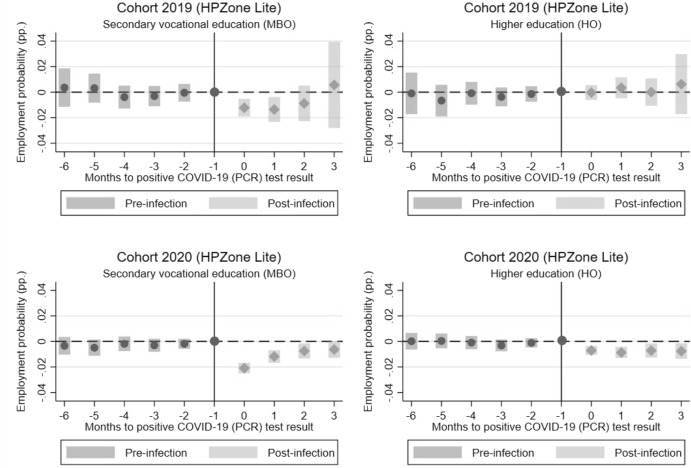
Event-study estimates of ATT by level of education (based on HPZone Lite). *Note*: The figure displays the event-study estimates (dots) and 95% confidence intervals (bars) of the ATT relative to the month of the first positive COVID-19 (PCR) test result for the 2019 and 2020 cohort based on HPZone Lite data. Period g-1 is used as the base-period and t as the post-period for ATT’s before (pre-infection) and after (post-infection) the infection takes place. *Source*: Statistics Netherlands, based on GGD HPZone Lite

**Fig. 8:**
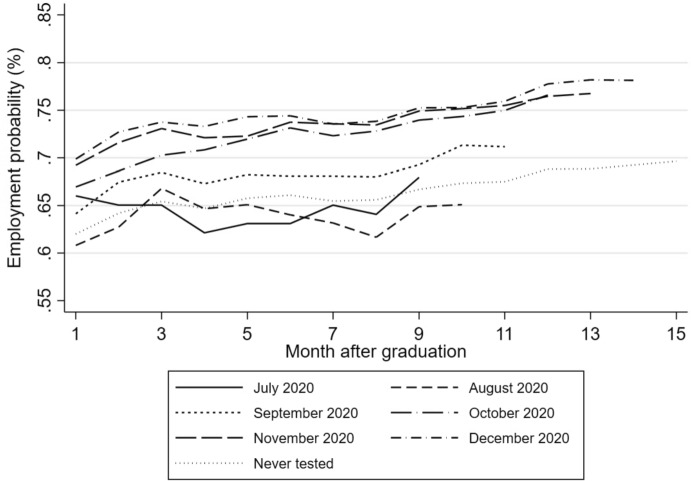
Pre-infection development of employment probabilities of the 2019 cohort by month after graduation and month of positive COVID-19 test result (based on HPZone Lite). *Note*: The figure displays the pre-infection development of employment probabilities of the 2019 cohort by month after graduation (1–15 months) and month of the first positive COVID-19 (PCR) test result (July 2020 till December 2020) based on HPZone Lite data. The pre-trend for the control group is reflected by the group with no registered COVID-19 (PCR) test results. *Source*: Statistics Netherlands, based on GGD HPZone Lite

Finally, we explore the heterogeneity of dynamic effects for graduates with a different level of education. We distinguish between secondary vocational (MBO) graduates and higher education (HO) graduates. The event-study estimates are displayed in Figs. 6 and 7 and are based on the CoronIT and HPZone Lite data. The estimated coefficients suggest that the drop in employment probabilities is larger for secondary vocational graduates than for higher education graduates. For the 2019 cohort who graduates from higher education, there is no estimated difference between control and treatment groups. As mentioned in Sect. 4.1, this is likely because these graduates are only observed in later periods and may have already found a job, making the impact of a COVID-19 infection smaller for them. For secondary vocational graduates, we observe a negative effect for both cohorts. Moreover, for secondary vocational graduates, the drop persists for longer period of time, compared to higher education graduates. A potential explanation is that the opportunity for remote work increases with the level of education making a period of quarantine less problematic for continuing to work. Since secondary vocational graduates more often work in occupations requiring physical presence at the workplace, they may find it less straightforward to work remotely in the event of a COVID-19 infection and especially during a period of quarantine. This could temporarily reduce their availability for work and lead to a drop in their productivity, which potentially lowers job prospects in the short term.

## Sensitivity analyses

The method we apply is potentially able to estimate unbiased treatment effects in our setting of dynamic treatment. To address the sensitivity of the method for the purpose of this paper, we conduct a number of checks. Table 3 presents the results of employing different estimation strategies to the data. The first set of checks is on our main ATT when estimated using several alternative estimation procedures (row A repeats the ATT from Table 2, Panel A). By conducting these checks, we assess the reliability of our estimated treatment effects and provide greater confidence that our results are not driven by specific modelling assumptions.

**Table 3:** Robustness check: Alternative estimation procedures. *Source:* Statistics Netherlands, based on GGD CoronIT and HPZone Lite

	2019 cohort		2020 cohort	
	(1) CoronIT	(2) HPZone Lite	(3) CoronIT	(4) HPZone Lite
A. Callaway and Sant’Anna (2021): improved doubly robust estimator	− 0.007*** (0.003)	− 0.005* (0.003)	− 0.011*** (0.002)	− 0.010*** (0.001)
B. de Chaisemartin & D’Haultfoeuille (2022): one time/one-way switching	− 0.007*** (0.003)	− 0.004 (0.003)	− 0.011*** (0.002)	− 0.011*** (0.002)
C. de Chaisemartin & D’Haultfoeuille (2022): multiple times/one-way switching	− 0.007*** (0.003)	− 0.004* (0.002)	− 0.011*** (0.002)	− 0.011*** (0.001)
D. de Chaisemartin & D’Haultfoeuille (2022): multiple times/two-way switching	− 0.007*** (0.003)	− 0.004 (0.003)	− 0.014*** (0.002)	− 0.013*** (0.002)
E. Borusyak et al. (2024): imputation estimator	− 0.003 (0.003)	− 0.002 (0.003)	− 0.008*** (0.002)	− 0.008*** (0.001)
F. Two-way fixed effects (TWFE) estimator	− 0.003 (0.003)	− 0.002 (0.002)	− 0.009*** (0.002)	− 0.008*** (0.001)

The table reports the group-time average treatment effects (ATT) of a COVID-19 infection on the employment probabilities of graduates from the 2019 (columns 1–2) and 2020 (columns 3–4) cohort. The coefficients are estimated using alternative (heterogeneity-robust) DID estimators and are based on data on COVID-19 (PCR) test results from the CoronIT (columns 1 and 3) and HPZone Lite (columns 2 and 4) database. Standard errors clustered at the individual level in parentheses; *p < 0.1, **p < 0.05, ***p < 0.01

First, we estimate the dynamic DID estimator developed by de Chaisemartin and D’Haultfoeuille (2022), which additionally allows for multiple treatment intensities and/or the treatment to switch on and off (rows B-D).^11^ We estimate three different specifications: one where the treatment variable remains switched on once infected (row B), consistent with our main analysis, one where the treatment variable also remains switched on once infected, but can increase in intensity with subsequent infections (row C), and one where the treatment variable switches off after one month but can switch on again if graduates are positively tested again (row D). The one-month period for switching off the treatment is not observed in the data but chosen as a modelling assumption to approximate the relative short duration of symptoms following a COVID-19 infection. Second, we estimate the imputation estimator developed by Borusyak et al. (2024) (row E), which imputes estimated non-treated potential outcomes using non-treated observations only to obtain an estimate of the treatment effect for each treated observation.^12^ Unlike Callaway and Sant’Anna (2021), which compares to the last pre-treatment period, Borusyak et al. (2024) use the average of all pre-treatment periods as the baseline. Finally, we estimate the conventional static TWFE estimator, including covariate-specific time fixed effects (row F). Unlike our main analysis, which includes only the never-infected in the control group, this specification treats both never-infected and not-yet infected individuals as control units.

The first two estimation procedures (Chaisemartin and D’Haultfoeuille 2022; Borusyak et al. 2024) potentially provide unbiased treatment effects in settings with multiple treatment periods and heterogeneous treatment effects like Callaway and Sant’Anna (2021), whereas the latter does not necessarily because of the earlier mentioned forbidden comparison problem. Almost all different estimation procedures generate an ATT of similar magnitude and statistical significance as our ATT in row A. Only the Borusyak-estimator and the TWFE estimator produce smaller and sometimes statistically insignificant results for the post-infection indicator. The deviating result from the Borusyak-estimator is most likely explained by the use of a different baseline, with our main analysis comparing to the last pre-treatment period, while this check uses the average of all pre-treatment periods. The deviating TWFE-result is most likely explained by the potential use of earlier infected individuals as controls for later infected individuals (i.e. ‘forbidden comparisons’) and differences in weighting procedures. Overall the results in Table 3 demonstrate that our findings are insensitive to alternative estimation procedures and are an improvement relative to the TWFE estimator.

Table 4 presents sensitivity analyses of our main results when estimated using alternative definitions of control and treatment groups. Conducting these robustness checks is important to ensure that our findings are not driven by the specific definition of control and treatment groups. This is especially important in this research because of the changing availability of COVID-19 tests during the pandemic. By systematically varying the definitions of control and treatment groups, we assess to what extent our estimated ATT remains consistent across different plausible specifications. The main aim is to provide a higher level of credibility of our estimated coefficients. Additionally, a placebo test helps to verify to what extent the estimated coefficients are likely to be due to a COVID-19 infection or that they may be driven by other factors, such as general trends in employment or selection into testing. If the ATT remains significant with a placebo treatment (e.g. a negative test), it suggests potential bias, while a disappearing effect supports the validity of our approach.

**Table 4:** Sensitivity analysis: Alternative control groups and treatment assignment. *Source:* Statistics Netherlands, based on GGD CoronIT and HPZone Lite

	2019 cohort		2020 cohort	
	(1) CoronIT	(2) HPZone Lite	(3) CoronIT	(4) HPZone Lite
A. Control group: negative testers and never testers	-0.007*** (0.003)	-0.005* (0.003)	-0.011*** (0.002)	-0.010*** (0.002)
B. Control group: only never testers	-0.007** (0.003)	-0.004 (0.003)	-0.010*** (0.002)	-0.010*** (0.002)
C. Control group: only negative testers	-0.008*** (0.003)	n/a	-0.011*** (0.002)	n/a
D. Control group: not-yet infected	-0.007*** (0.003)	-0.005* (0.003)	-0.011*** (0.002)	-0.010*** (0.001)
E. Treatment: negative COVID-19 test result compared to never testers	0.002* (0.001)	n/a	0.002* (0.001)	n/a
F. Treatment: negative COVID-19 test result compared to positive testers	0.001 (0.002)	n/a	-0.001 (0.002)	n/a

The table reports the group-time average treatment effects (ATT) of a COVID-19 infection on the employment probabilities of graduates from the 2019 (columns 1–2) and 2020 (columns 3–4) cohort using alternative definitions of the control and treatment group. The coefficients are estimated using the heterogeneity-robust DID estimator by Callaway & Sant’Anna (2021) and are based on data on COVID-19 (PCR) test results from the CoronIT (columns 1 and 3) and HPZone Lite (columns 2 and 4) database. N/a means that there are no data available on negative COVID-19 test results in the HPZone Lite data in order to use this to define a alternative control group or treatment assignment. In all cases, the size of the control and treatment group remain large enough for statistical inference. Standard errors clustered at the individual level in parentheses; *p < 0.1, **p < 0.05, ***p < 0.01

Row A copies the estimated coefficients from Table 2 Panel A. Rows B-F present the estimated coefficients from several different definitions. The results suggest that the ATT is not affected by using only individuals who never test (row B), only individuals with a negative test results (row C) and only not-yet infected individuals (row D) as control groups. Finally, the results suggest that the negative ATT disappears (or turns into a small positive effect) when using a negative COVID-19 test result instead of a positive COVID-19 test result as a placebo treatment (rows E and F). For negative testers there is a relatively large anticipatory effect at $t-1$, since having a job is positively associated with testing. Compared to positive testers (row F), we do not observe any significant effect. All in all, the estimated coefficients seem to confirm the robustness of our main findings to alternative definitions of the control groups and treatment variable.

## Discussion and conclusion

This research documents and interprets the effect of COVID-19 infections on employment probabilities of labour-market entrants in the Netherlands. The empirical strategy exploits the variation in (positive) COVID-19 test results registered at Municipal Health Services (GGD) among individuals over time, by making use of a heterogeneity-robust difference-in-difference model. The estimated coefficients suggest that a COVID-19 infection temporarily decreases the employment probabilities of positively tested labour market entrants (ATT) by 0.5–1.1 percentage points on average over a three-month period within the first fifteen months after graduation. The results are robust to different models and sensitivity analyses suggest that different definitions of control and treatment groups do not alter the estimated effects. The magnitude and duration of the employment effect is relatively limited and short-lived. Graduates from secondary vocational education and individuals with the lowest labour-market tenure seem to be most affected by contracting COVID-19.

The size of the estimated coefficients is most likely an underestimate of the real effects of contracting COVID-19 on employment because some infected graduates are misclassified as part of the control group, particularly during periods of high infection rates when asymptomatic or untested cases were more common. Our database—which is unique because it contains test results at the individual level—only contains individuals who have been tested and registered by the Municipal Health Services (GGD). Testing by these services is limited because of capacity constraints and many people use different tests (other than PCR tests) and test at home. Positive tests of those individuals are not recorded and these people are likely to be part of the control group in our data. If the effect of a positive test does not differ between different tests, our results can be viewed as a lower bound of the impact of contracting COVID-19 on employment probabilities. A potential limitation of our work is that we have not been able to discriminate between the effects of a quarantine period, which has to be taken into account, and the severeness of contracting COVID-19 in terms of becoming ill. If people only face mild symptoms from contracting COVID-19, employment effects are likely to be small, if labour-market entrants become ill for a substantial period the effect on finding a job might be larger. Given the evidence in the medical literature that adolescents and young adults are on average less likely to become severely ill, it is more likely that we estimate the effect of a forced period of quarantine after contracting COVID-19 on employment probabilities.

Finally, the results confirm that using a heterogeneity-robust DID estimator likely results in less biased estimates compared to estimates derived from the convential TWFE estimator, which might suffer from the ‘forbidden comparison’ problem. The results seem largely robust to using different heterogeneity-robust estimators, which strengthens the internal validity of the results.

## Figures and Tables

**Fig. 9:**
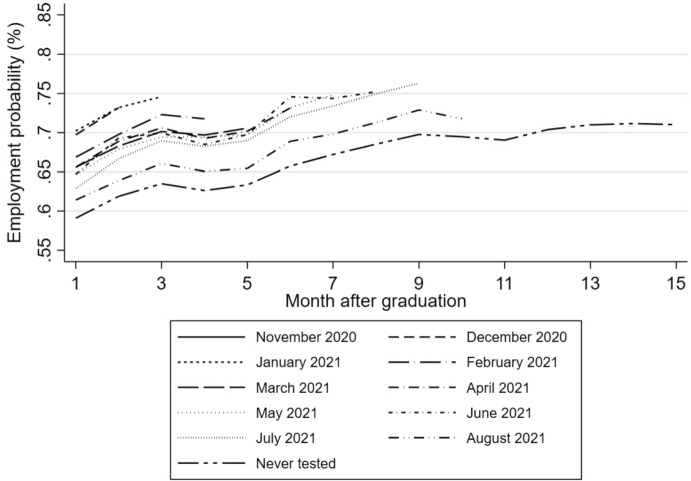
Pre-infection development of employment probabilities of the 2020 cohort by month after graduation and month of positive COVID-19 test result (based on HPZone Lite). *Note*: The figure displays the pre-infection development of employment probabilities of the 2020 cohort by month after graduation (1–15 months) and month of the first positive COVID-19 (PCR) test result (November 2020 till August 2021) based on HPZone Lite data. The pre-trend for the control group is reflected by the group with no registered COVID-19 (PCR) test results. *Source*: Statistics Netherlands, based on GGD HPZone Lite

## Appendix

See Appendix Figs. 8, 9 and Tables

**Table 5:** Summary statistics (based on GGD CoronIT). *Source*: Statistics Netherlands, based on GGD CoronIT

	Cohort 2019			Cohort 2020		
	Infected	Never-infected	Difference	Infected	Never-infected	Difference
*Outcome variable*						
Employment probability (t = 1)	0.717	0.697	0.020***	0.701	0.675	0.026***
*Socio-economic variables*						
Female (yes = 1)	0.529	0.506	0.023***	0.515	0.509	0.007**
Age (years)	22.986	23.087	− 0.101***	22.801	23.148	− 0.347***
Migration background (yes = 1)	0.270	0.197	0.073***	0.220	0.196	0.024***
Higher education degree (yes = 1)	0.484	0.482	0.003	0.480	0.496	− 0.016***
Higher educated parents (yes = 1)	0.271	0.296	− 0.025***	0.292	0.310	− 0.018***
Household members (yes = 1)	0.816	0.805	0.011***	0.823	0.805	0.018***
Health issues (yes = 1)	0.217	0.215	0.002	0.205	0.213	− 0.008***
No. of graduates	9,427	150,959		23,293	130,421	

^***^*p* < *0.1, **p* < *0.05, ***p* < *0.01*

^5^,

**Table 6:** Summary statistics (based on GGD HPZone Lite). *Source**:* Statistics Netherlands, based on GGD HPZone Lite

	Cohort 2019			Cohort 2020		
	Infected	Never-infected	Difference	Infected	Never-infected	Difference
*Outcome variable*						
Employment probability (t = 1)	0.728	0.697	0.031***	0.706	0.670	0.037***
*Socio-economic variables*						
Female (yes = 1)	0.551	0.504	0.047***	0.535	0.501	0.034***
Age (years)	22.983	23.088	− 0.105***	22.884	23.165	− 0.281***
Migration background (yes = 1)	0.264	0.197	0.067***	0.220	0.193	0.027***
Higher education degree (yes = 1)	0.483	0.482	0.001	0.471	0.501	− 0.030***
Higher educated parents (yes = 1)	0.271	0.296	− 0.025***	0.288	0.313	− 0.025***
Household members (yes = 1)	0.816	0.805	0.012***	0.819	0.804	− 0.015***
Health issues (yes = 1)	0.223	0.214	0.008**	0.209	0.213	− 0.005**
No. of graduates	10,474	149,912		37,756	115,958	

^6^.

## Footnotes

1 Bussink et al. (2022) show that the short-term employment probability of graduates in the Netherlands deteriorated by up to 4 percentage points in 2020 (from a level of 77 percent).

2 COVID-19 is caused by the coronavirus SARS-CoV-2. The disease leads to respiratory problems (breathing problems) and fever. The virus is spread by droplets released when coughing, sneezing or talking. Infection occurs because these droplets end up in the mouth, nose or eyes.

3 This definition of labour-market entrants is similar to the definition used by Statistics Netherlands (CBS) and has been used in previous research as well. See Bisschop et al. (2020) and Bussink et al. (2022). The year of entering the labour market after completing education is defined as having obtained a diploma and being no longer registered in education by the first of October.

4 The results do not significantly change if we exclude graduates whose COVID-19 test results are inconclusive or unknown from the analysis.

5 This finding is corroborated by the estimated coefficients in Bussink et al. (2022). They obtain estimates suggesting a large effect on employment of lockdown for the 2019 cohort relative to the 2020 cohort. Our findings suggest a larger effect for the 2020 cohort, which we measure closer to labour-market entrance.

6 Life satisfaction among young people seems to have dropped during the economic lockdowns, which could be related to lower job satisfaction among young workers (Neugebauer et al. 2023).

7 A polymerase chain reaction test (PCR test) takes a sample of ribonucleic acid (RNA) and amplifies it with the help of lab technologies. Amplifying RNA helps to make even small traces of the COVID-19 virus visible in the test sample. Even if individuals have a small trace of the virus in their system, the PCR test will detect it.

8 There is a reporting obligation in the Netherlands for certain infectious diseases that also applies to COVID-19 (so-called category A diseases). The reporting obligation requires that doctors and medical microbiological laboratories report certain infectious diseases to the Municipal Health Service (GGD).

9 In particular, compared to the estimator of Borusyak et al. (2024), which may be preferable in settings where the outcome is not too serially correlated and confidence in parallel trends across all periods is high. The Borusyak-estimator imposes parallel trends for all groups and time periods, whereas the Callaway and Sant’Anna-estimator only relies on post-treatment parallel trends (see Roth et al. 2023).

10 All estimates are computed by the *csdid* Stata command (see Rios-Avila et al. 2023).

11 These estimates are computed by the *did_multiplegt* Stata command (Chaisemartin et al. 2019).

12 These estimates are computed by the *did_imputation* Stata command (Borusyak 2023).
